# Memory in Spina Bifida, from Childhood to Adulthood: A Systematic Review

**DOI:** 10.3390/jcm13175273

**Published:** 2024-09-05

**Authors:** Imanol Amayra, Aitana Ruiz de Lazcano, Monika Salgueiro, Samuel Anguiano, Malena Ureña, Oscar Martínez

**Affiliations:** 1 Neuro-E-Motion Research Team, Department of Psychology, Faculty of Health Sciences, University of Deusto, 48007 Bilbao, Spain; a.ruizdelazcano@deusto.es (A.R.d.L.); sam.anguiano@opendeusto.es (S.A.); oscar.martinez@deusto.es (O.M.); 2 Department of Clinical and Health Psychology and Research Methodology, Faculty of Psychology, University of the Basque Country UPV/EHU, 20018 Donostia, Spain; monika.salgueiro@ehu.eus; 3 Independent Researcher, 48970 Basauri, Spain; malena@opendeusto.es

**Keywords:** spina bifida, myelomeningocele, cognitive functioning, memory, children, adolescents, adults

## Abstract

**Background:** Spina bifida (SB) is a rare congenital disease characterized by not only physical but also neuropsychological disturbances. Among these neuropsychological impairments, memory deficits are a significant concern, as they substantially hinder aspects of crucial importance in the lives of individuals with SB such as medical needs or daily life activities. The main objective is to conduct a systematic review of the current evidence on the memory deficits in the SB population, including children, adolescents, and adults. **Methods:** Four databases (PubMed, SCOPUS, Web of Science, and ProQuest) were systematically screened for eligible studies. **Results:** The present review reveals cognitive difficulties in different memory types among individuals with SB. These deficits, identified in childhood, seem to persist into adulthood. Specifically, impairments are evident in short-term memory, working memory, and long-term memory. The neuropsychological instruments applied in the studies that were included in this systematic review vary, however, most reach the same conclusions. **Conclusions:** The present findings underscore the importance of incorporating cognitive assessments, particularly those focused on the memory domain, into routine childhood evaluations for individuals with SB. Early identification of these cognitive difficulties allows for the timely implementation of cognitive interventions that could leverage the inherent plasticity of the developing brain, and prevent or delay the onset of these deficits in later adulthood for people with SB, ultimately improving their functionality and quality of life.

## 1. Introduction

Spina bifida (SB) is one of the most common neural tube defects [1,2,3] compatible with a lengthy lifespan [4]. This congenital malformation presents with a wide range of anomalies affecting the spinal cord, brain, brainstem, and peripheral nerves [5,6,7]. As a result, SB causes varying degrees of long-lasting effects in various systems and functions, from childhood to adulthood, including physical and cognitive impairments [8,9,10,11,12]. Due to these diverse and complex lifelong complications, individuals with SB require close monitoring and comprehensive treatment [13]. In addition to the aforementioned challenges, individuals with SB often experience stigma [14,15,16], social discrimination [16,17,18], social participation restrictions [19], poorer academic and vocational outcomes [20,21,22], and psychological difficulties [13,23,24,25,26,27,28,29], placing them at a higher risk of maladjustment [30].

As children with SB age, their associated difficulties often worsen, leading to a decline in overall health [31,32]. The challenges of adulthood, compounded by physical and neurological limitations, render independent living increasingly arduous [33,34,35]. Managing chronic complications and the disease itself negatively impacts the quality of life for individuals with SB [36,37,38,39]. Consequently, they exhibit lower quality of life compared to both healthy peers and individuals with other chronic conditions [31,37,39].

One of the most challenging consequences for individuals with SB is the emergence of cognitive deficits [40,41,42], which can vary in severity [43,44] and become more pronounced in adolescence and adulthood [43]. The cognitive profile of patients with SB remains an under-researched area [43] due to the challenges of studying a rare disease. Furthermore, the presence of variable intellectual outcomes [11,12,45,46,47] or comorbidities (such as hydrocephalus, Arnold Chiari malformation, or corpus callosum dysgenesis) [31,48,49,50,51,52,53,54] complicates the isolation of cognitive deficits solely attributable to SB [54].

Despite these challenges, several studies have examined various cognitive domains in the SB population, generating significant interest. Findings consistently indicate that individuals with this diagnosis often exhibit difficulties in language [47,55,56], numeracy [57,58,59,60], executive function [61,62,63,64,65,66,67], attention [63,66,68,69,70,71,72,73], and visuospatial skills [43,57,70]. Beyond the cognitive domains previously mentioned, memory is another key cognitive are of impairment for individuals with SB.

Memory is crucial for developing and maintaining cognitive and academic skills across the lifespan [56]. While memory decline is a normal part of aging [74], individuals with SB often experience memory difficulties earlier in life compared to their peers [75,76]. Furthermore, these memory deficits appear to progress more rapidly in adults with SB [56]. Moreover, specific memory types, including prospective [77,78,79], working [80], and verbal memory [81,82], are essential for daily life. Working memory, particularly impaired in SB [7,8,9,10,11,12,13,14,15,16,17,18,19,20,21,22,23,24,25,26,27,28,29,30,31,32,33,34,35,36,37,38,39,40,41,42,43,44,45,46,47,48,49,50,51,52,53,54,55,56,57,58,59,60], is crucial for academic success, especially in mathematics [83]. Retrospective memory deficits in this population are associated with decreased independence, communication difficulties, and lower quality of life [77]. Therefore, identifying specific memory deficits in individuals with SB is crucial as they represent unmet needs with significant downstream consequences for academic achievement and job opportunities [57], daily activities [42], and quality of life [59,60,61,62,63,64,65,66,67,68,69,70,71,72,73,74,75,76,77,78,79,80,81,82,83,84].

Although the underlying causes of these memory impairments remain unclear [56], ongoing research suggests potential associations with hydrocephalus [54], Chiari malformation [54], and structural abnormalities of the brain. These anomalies include alterations in hippocampal volume [85,86], atypical development of the frontal lobe [85], and diminished white matter integrity within the uncinate fasciculus [87]. Further research is necessary to elucidate the complex interplay between these factors and their impact on memory in the SB population.

Given these factors, it seems clear that adults with SB may require cognitive therapies, retraining, or support focused on the memory domain earlier in life, rather than waiting until memory deficits become apparent and limit independence [56]. To this end, prompt identification of memory deficits is essential to mitigate secondary conditions and support individuals in achieving adult milestones [88]. Certain cognitive difficulties are particularly noteworthy as they impact both medical care and functional independence of patients with SB, ultimately leading to a greater reliance on caregivers [84].

Despite the critical importance of understanding memory deficits in individuals with SB, a comprehensive systematic review examining this topic across the pediatric, adolescent, and adult populations remains conspicuously absent from the literature. To address this knowledge gap, this study will conduct a systematic review of existing research on memory cognition assessment in SB patients across the lifespan. A comprehensive understanding of the specific memory needs of this population will enable health professionals to design and develop targeted interventions that effectively address their unique challenges, possibly resulting in a higher quality of life.

## 2. Materials and Methods

This review has been conducted considering the PICOS criteria (Table 1) and following the PRISMA (Preferred Reporting Items for Systematic Reviews and Meta-Analysis) methodology [89]. The study protocol has been registered in the Open Science Framework (OSF) (Associated project: osf.io/8jwf4).

### 2.1. Eligibility Criteria

The inclusion and exclusion criteria for the present systematic review are presented in Table 2.

### 2.2. Search Strategy

A literature search was performed in order to identify empirical studies that used cognitive tests to measure the functioning in different types of memory in patients with SB. We queried the following online databases: PubMed, SCOPUS, Web of Science, and ProQuest. The search terms used were (a) spina bifida, (b) myelomeningocele, and (c) memory. The search was designed to capture all results containing any combination of these keywords (Table 3). The literature search was conducted until 1 July 2024.

### 2.3. Data Extraction

**Figure 1:**
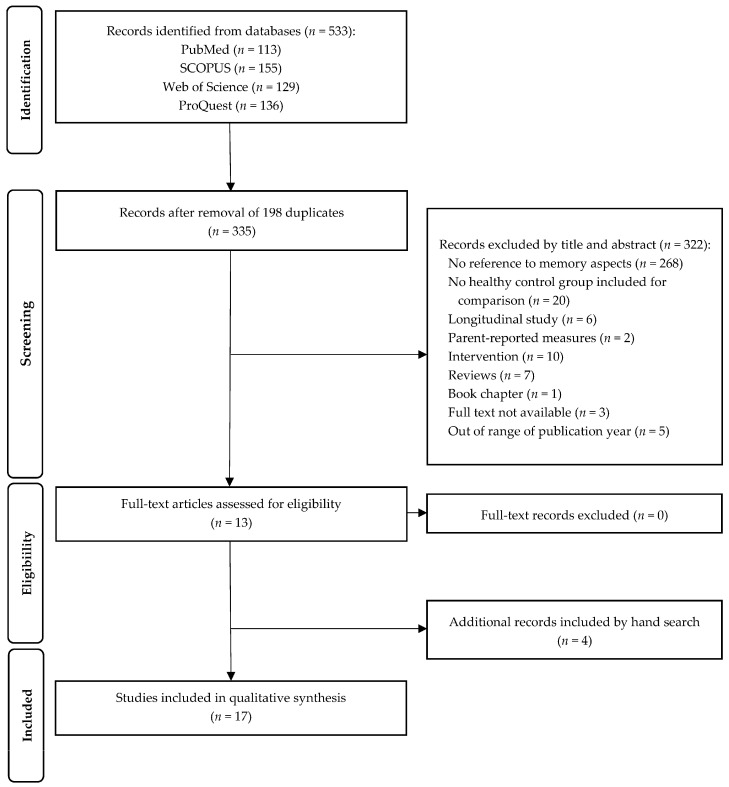
A PRISMA flow diagram of the search strategy for cognitive studies assessing memory in patients with SB.

A preliminary search identified a total of 533 studies (A.R.d.L.). After removing 198 duplicates, 322 were screened by two independent authors (A.R.d.L. and M.U.) analyzing the titles and abstracts. When there was a disagreement, a third reviewer helped to achieve a consensus (I.A.). After the screening and reviewing for inclusion and exclusion criteria, a total of 13 studies were selected for the full review (A.R.d.L. and M.U.). Additionally, 4 articles were included by manual search. In total, 17 papers were included in this systematic review. The detailed selection process is illustrated in Figure 1.

The following data were extracted from the articles:

- The authors and year of publication;
- Sample (experimental and control);
- Demographic variables (age and sex);
- Outcomes on the neuropsychological memory assessments;
- Cognitive tests and/or tasks used to measure memory;
- Subtests applied;
- Type of memory assessed.

### 2.4. Risk of Bias

The adapted version of the modified Newcastle–Ottawa Scale was used to assess the risk of bias in each study included [90]. This scale permits evaluation of the evidence quality in each publication by assessing the following aspects: participant selection (selection bias), control confounding (performance bias), statistical methods (detection bias), and outcome measures (information bias).

## 3. Results

### 3.1. General Overview

Out of the original 533 articles that were considered, 17 studies were deemed eligible for inclusion in this systematic review. Among these selected studies, 12 focused on pediatric and adolescent populations, while the remaining 5 were conducted with adult samples.

Analysis of study quality and potential biases of the eligible articles revealed shortcomings in sample-related methods. To strengthen future research, these methods should be enhanced. Key challenges for improvement include the need for larger participant groups (Table 4).

From a global view, the majority of the studies included in the review were conducted with participants with myelomeningocele (the most common subtype of SB) [86,91] with high- and low-level lesions. Only one study, by Iddon et al. [98], did not specify the type of SB diagnosed in its participants. Conversely, all studies included in the present review involved samples of participants diagnosed with hydrocephalus, except for that of Iddon et al. [97], where an additional group of individuals with SB, but without hydrocephalus, was added for comparison with those with both SB and hydrocephalus. Similarly, many of the included articles comprised samples with a diagnosis of Arnold Chiari II malformation. However, it is important to note that some studies did not specify this information [49,63,76,77,86,91,92,93,94,96,97,98,99].

The seventeen studies included in the present systematic review reported at least one type of decreased memory in SB patients. Nine of the selected articles included sample sizes exceeding 30 SB participants [60,63,86,91,94,95,97,99,100]. In contrast, the remaining studies had sample sizes with fewer than 30 participants.

Furthermore, regarding the cognitive assessments employed in the included studies, there is significant variability. As a result, few tests were repeated across studies. In the pediatric and adolescent population, the only tests repeated among the selected studies were the CBT to assess visuospatial WM [76,93,97], the “Numbers Reversed” subtest of the WJ-R battery [60,63], and the “Digit Span” subtest from the WISC battery [49,91,93], both assessing WM. Conversely, in the adult population, several subtests of the RBMT-E battery were used repeatedly across studies. Specifically, the “Belongings”, “Appointments”, and “Messages” subtests were employed in two studies [77,86] to assess prospective memory, while the “Immediate Story” and “Delayed Story” subtests evaluated episodic verbal memory.

Due to this heterogeneity, direct comparisons between different research findings are challenging, making it difficult to draw clear conclusions. To provide a clearer overview of the neuropsychological instruments and cognitive tasks used in the included studies, detailed lists are presented in Table 5 (for child and adolescent SB populations) and Table 6 (for adult SB population).

### 3.2. Memory Functioning in Children and Adolescents with SB

Among the articles that included both pediatric and adolescent populations, 12 studies met the established criteria (Table 7). Participants ranged in age from 7 to 18 years old.

All studies included a control group of typically developed children and adolescents. Furthermore, three studies incorporated additional clinical groups (consisting of patients with an AMC, hydrocephalus, or AS diagnosis) for comparison of cognitive outcomes [93,95,97]. As detailed in Table 7, six out of twelve studies included more than 30 patients with SB [60,63,91,94,95,97], while the other six studies had fewer participants.

Seven of the studies reviewed employed age- and gender-matched control groups [49,75,91,92,93,95,96]. The remaining studies were either age-matched [60,76,94] or gender-matched [63,97], but not both. Socioeconomic status was taken into account in some of the studies included in the present review [60,75,95]. Only one of the twelve studies reviewed did account for participants’ handedness [93]. Just the study carried out by Mammarella et al. [76] accounted for years of education. Some other studies accounted for parental education [75,91].

With regard to the SB pediatric and adolescent patients’ performance on the neuropsychological tests and tasks that assessed memory, the present review suggested a generalized deficit in this population. Firstly, impaired working memory performance was reported in SB patients [49,63,91]. However, three studies using the “Digit Span” subtest from the WISC-II or WISC-IV neuropsychological batteries found no significant differences between SB patients and healthy controls [91,93,96]. Similarly, as noted by Boyer et al. [91], the “Arithmetic” subtest from the WISC-III, also used to assess working memory, is not appropriate due to its reliance on mathematical skills, which are impaired in the SB population. Consequently, caution should be exercised when interpreting results from this subtest.

Continuing with the results obtained on working memory, specific deficits in verbal [60,96], visual [76], spatial [92], topographic [97], and visuospatial [92,96,97] working memory were identified in children and adolescents with SB ranging from 7 to 16 years old. Nevertheless, a consensus has not been reached by all authors, as a few [76,93] have failed to identify some of the aforementioned working memory deficits.

While several previously mentioned studies [92,96,97] identified spatial working memory difficulties in children and adolescents with SB, Mammarella et al. [76] and Lindquist et al. [93] did not find these deficits, even when using the same cognitive task (“Corsi block task”) to measure it as Bartonek et al. [97]. Consequently, the participants with SB in their study did not exhibit significant differences from the control group in tasks requiring spatial working memory [76]. However, they did observe a poorer performance on a visual working memory task [76]. Because of this, the authors suggest that children seem to exhibit difficulties in tasks believed to rely on passive cognitive processes, whereas their performance is comparable to typically developing children on tasks thought to involve more active visual working memory components [76].

Interestingly, Raghubar et al. [60] demonstrated that verbal working memory significantly mediated group differences in arithmetic task performance. Similarly, visuospatial working memory mediated group differences in math fluency [60]. The results obtained by Attout et al. [96] add additional information to the above. Thus, their results reflect how the fundamental numerical challenges observed in individuals with SB might not stem from a basic inability to represent numbers [96]. Instead, these difficulties could be the results of broader cognitive problems, particularly weaknesses in working memory [96].

Regarding the visuospatial working memory tasks applied in the study by Bartonek et al. [97], non-ambulant infants or adolescents with SB, who required assistance for mobility, appeared to exhibit a poorer performance. These findings were also replicated in the study by Jansen-Osmann et al. [92], who employed a virtual maze that allowed the assessment of spatial memory in the youngest patients with SB. In this way, it was observed that children with SB, who had a later onset of walking, demonstrated lower scores and, consequently, poorer performance on spatial memory, requiring more attempts to learn the correct way through the virtual maze [92]. Controlling for cognitive differences observed in Jansen-Osmann et al. [92], the poorer performance of children with SB in the virtual maze was attributed to their restricted mobility.

Difficulties in short-term and long-term visuospatial memory were identified in one of the studies reviewed through the application of the Rey-Osterrieth Complex Figure Test [93]. Children with SB and with ages ranging from 8 to 13 years old demonstrated significant impairments in visuospatial memory compared to healthy controls during the performance of this test, as evidenced by their greater difficulty in drawing the figure [93].

Verbal memory, specifically short-term [93] and explicit memory [63,75,93,95], appeared to be another type of memory affected in children and adolescents with SB, ranging in age from 7 to 18 years old. Due to these difficulties, young people with SB recalled fewer words in comparison to their healthy peers. Conversely, Attout et al. [96] found no significant differences between the SB group and the healthy control group, observing a similar performance on a short-term memory task.

In reference to studies demonstrating verbal memory deficits in the SB population, such as that by Vachha and Adams [75], individuals with SB struggled to identify optimal learning strategies for the word lists to maximize performance on verbal learning tasks. Hampton et al. [95] compared a clinical group with SB to another with aqueductal stenosis. The latter group shared a clinical similarity with the SB population: a hydrocephalus diagnosis. However, the SB group exhibited significantly lower scores on the verbal memory task than the other clinical group [95]. These findings suggest that factors beyond hydrocephalus, likely related to the more severe brain abnormalities in SB, contributed to the observed disparities [95].

In addition, in the study by Burro et al. [49], additional subtests were employed to assess cognitive domains beyond the scope of this systematic review, such as processing speed. These subtests demand strong visual short-term memory and visual sequence memory, among other abilities [49]. Given the Processing Speed Index’s low mean scores, difficulties in these memory areas might also be present [49], necessitating further research to confirm this hypothesis. A similar situation arises with the Perceptual Reasoning Index, where one of the subtests used to measure this cognitive domain, “Matrix Reasoning”, also requires visual short-term memory skills [49].

### 3.3. Memory Functioning in Adults with SB

In the present systematic review, five studies met the inclusion (Table 8). The ages of the participants ranged from 18 to 62 years old.

Every study included a control group made up of normally developing adults. As shown in Table 8, only one study included fewer than 30 adult participants with SB [77]. The sample size was not specified in the study by Iddon et al. [98], and varied depending on the neuropsychological test. Only one study incorporated additional clinical groups (consisting of patients with hydrocephalus alone and patients with SB alone without concomitant hydrocephalus) for comparison of cognitive outcomes [98].

Three of the studies [77,86,99] employed age- and gender-matched control groups, revealing no differences between groups regarding these variables. However, while participants in Ware et al. [100] were age-matched, significant gender differences were observed, with a higher proportion of males in the clinical SB group compared to the healthily developing peers group. The information concerning age or gender differences between the groups is not detailed in the study by Iddon et al. [98]. Two of the reviewed studies [86,100] accounted for the participant handedness, but found no statistically significant differences between the clinical SB group and the control group. Only two studies accounted for the socioeconomic status of their participants [86,100], and none of the studies paired their sample taking into account the participants’ years of education or educational levels.

Adults with SB also exhibited memory deficits, as indicated by the results of the neuropsychological tests and tasks. These included deficits previously identified in children and adolescents, as well as impairments in memory types not previously assessed in the younger populations. These findings suggest the potential for enduring memory challenges in individuals with SB throughout their lifespan, although further research is needed to confirm this hypothesis.

In this way, similar to pediatric and adolescent populations, adults with SB exhibited visuospatial [98] and verbal memory deficits [98], particularly in tasks involving verbal episodic memory [77,86,100]. These difficulties in episodic memory were not associated with lower hippocampal volumes in the participants from Treble-Barna et al. [86].

General [77] and specific working memory deficits, including visual [100] and spatial working memory [98,100], were also identified in adults with SB. In reference to reaction times during the working memory tasks, this was only taken into account by Ware et al. [100]. In this way, reaction times on the visual working memory tasks did not differ between the SB sample and their healthy peers [100]. Shunting had no impact on these working memory results [100]. By applying neuroimaging techniques, it could be observed how reduced hippocampus volume in the SB group was associated with slower responses during the visual working memory task [100]. On the other hand, the volume of the dorsolateral prefrontal cortex was inversely related to the response speed for information maintained during the working memory task [100]. Regarding the results obtained specifically in Dennis et al. [77], a more effective performance was observed in working memory tasks demanding inhibitory control and rule-following, but a weaker performance in tasks requiring the retention and manipulation of large amounts of information. In addition, working memory function correlated positively with communication and community living independence in one of the studies [77].

Prospective memory, a memory type not assessed in studies of the youngest population, was evaluated in adults with SB. Thus, prospective memory deficits were identified in adults with SB [77,86,99], both in time-based and event-based tasks [99]. Participants in Dennis et al. [77] with more shunt revisions exhibited poorer prospective memory compared to those with fewer revisions. However, even participants with fewer revisions demonstrated memory deficits, despite these findings [77]. At the same time, while these prospective memory difficulties were not related to shunt history [99], they were associated with the significantly smaller hippocampal volumes observed in the participants and some aspects of their functional independence [86].

No deficits were observed in non-declarative or semantic memory among adults with SB. A significant finding was that adults with SB and fewer than six shunt revisions outperformed the normative population in non-declarative memory [77].

Intra-group differences were observed in the performances of adults in the SB clinical group. On the one hand, SB patients with more than five shunt revisions seemed to have a worse performance on both prospective memory and verbal episodic memory tasks than those with fewer shunt revisions [77]. The results obtained by Iddon et al. [97] revealed a more severe pattern of impairment in SB participants with hydrocephalus compared to SB adults without concomitant hydrocephalus. On the other hand, older adults with SB had more difficulties during the prospective memory task than the younger adults [98]. Nevertheless, this difference is not replicated in the study where visual WM was evaluated, observing a similar performance between the youngest and the oldest adults with SB [99].

## 4. Discussion

To the best of our knowledge, no comprehensive analysis has been conducted to critically assess evidence of memory impairments in SB. Therefore, we conducted a systematic literature review of potential memory deficits in pediatric, adolescent, and adult patients with SB. A total of 12 studies involving pediatric and adolescent populations, and five studies involving adult populations diagnosed with SB, were reviewed. Consequently, the existing literature was divided into pediatric and adolescent studies (Table 7), and adult studies (Table 8) assessing memory in SB patients.

The present systematic review found limited empirical evidence investigating memory impairments in the SB population. While the number of studies focusing on children or adolescents and adults was uneven, both groups exhibited cognitive challenges related to the memory domain across all included studies. There appears to be a general consensus that short-term memory [63,86,93,95,98], working memory [49,60,63,76,77,91,92,94,96,97,98,100], and long-term memory [63,75,77,86,93,95,98,99] (specifically, declarative memory) are the most affected subtypes of memory in the SB population. Within the observed long-term memory difficulties, both retrospective and prospective memory appear to be affected in the SB population.

As observed in the reviewed articles, declarative memory has been a significant focus, with shared deficits identified among SB patients. Despite this emphasis, procedimental memory has been overlooked. None of the reviewed studies investigated potential procedural memory deficits in individuals with SB. Given the importance of procedural memory in daily living and the challenges faced by SB patients in these activities, investigating this memory type is crucial for future studies.

Conversely, non-declarative memory does not appear to be affected in the SB population [77]. However, due to the identified risk of bias in our analysis, primarily related to participant selection and sample size limitations, establishing a definitive profile of memory deficits associated with SB is challenging.

It is important to note that this review exclusively included studies of patients with myelomeningocele-type SB, most of whom also had comorbid hydrocephalus and Arnold Chiari II malformation. These were the only studies meeting our inclusion criteria. Consequently, research on memory in other SB subtypes, with or without comorbid diagnoses, was excluded (for example, Barf et al. [61]), as they did not meet our criteria. Future studies comparing cognitive performance across different SB subtypes (with and without hydrocephalus and Chiari II), and healthy controls, are necessary to identify potential subtype differences.

Furthermore, a significant heterogeneity in neuropsychological assessment protocols was observed among reviewed studies, hindering definitive conclusions about memory. Additionally, the influence of variables like anxious-depressive symptoms, pain, and sleep quality was uncontrolled across studies. Given the potential relationship between these factors and cognitive performance, they should be considered in future research.

As previously noted, some studies involved small sample sizes and no longitudinal studies were included in this systematic review. Consequently, interpretations and conclusions about the cognitive function, specifically memory, of the SB population should be approached cautiously. Additionally, seven of the sixteen reviewed studies lacked a comparative group matched for age and gender, and all but one pediatric and adolescent study [76] did not account for participants’ years of education. Importantly, several articles analyzing memory were excluded due to their lacking a control group for comparison (Figure 1). Future studies should address these methodological limitations.

Comparing this systematic review with a previous work by Lindquist et al. [101], which summarized various cognitive domains, including memory, reveals a lack of substantial progress in SB cognitive research, particularly in memory. The latest studies addressing memory in the SB population and meeting our inclusion criteria were published in 2020 [96] and 2021 [97], focusing on pediatric and adolescent populations. No further studies meeting our criteria have been published since 2017 for adults with SB [100].

Studies have revealed brain structure abnormalities in SB patients compared to healthy controls. Beyond identifying Chiari II malformation [86,95,100], smaller corpus callosum [94,98,102], hippocampus [86,100,103], posterior fossa and cerebellar volumes [104], and larger putamen, superior parietal cortex, dorsolateral prefrontal cortex [100], and amygdala [86] volumes have been reported. Ware et al. [100] observed accelerated dorsolateral prefrontal cortex volume reduction in SB adults, mirroring typical age-related changes but at a faster pace. Additionally, these adults exhibited reduced superior parietal cortex volume compared to both typical aging and healthy controls. Conversely, thalamus and caudate nucleus volumes remained relatively stable in SB adults [100]. However, longitudinal studies are essential to definitively validate the accelerated aging hypothesis in SB patients [86]. Three studies in the present systematic review used neuroimaging to support cognitive findings: one pediatric/adolescent [95] and two adult studies [86,100]. Two studies found significant associations between cognitive performance and volumetric measures [86,100]. Nonetheless, these associations require confirmation through comprehensive studies incorporating extensive cognitive protocols, longitudinal assessments, and neuroimaging.

Elevated rates of anxious-depressive symptomatology have been reported in both pediatric and/or adolescent [29,105,106,107] and adult [23,25,27,108] SB populations. Bellin et al. [23] found that nearly half (49.2%) of young adults (18–25 years) reported clinical-level psychological difficulties. Among them, 53.3% exhibited both depressive and anxiety symptoms, 33.3% within the clinical range for depressive symptoms only, and 13.3% within such a range for anxiety symptoms only. Showen et al. [108] reported that over half (56%) of individuals with SB (18–72 years) experienced depression, anxiety, or both. Anxiety severity ranged from mild (12%) to moderate (28%) to severe (7%), while depression ranged from mild (14%) to moderate (29%) to severe (5%). While these data are available for adult populations, rates for pediatric populations remain unreported.

Additionally, SB patients experience fatigue [27], high levels of pain [109,110], and poor sleep efficiency [111], including sleep-related breathing disorders [112]. Recognizing neurocognitive and psychological symptoms, as well as factors like fatigue, pain, and sleep quality, is crucial for understanding and addressing the complexities faced by these patients.

Lastly, it is crucial to highlight the limitations of the studies examined in this systematic review. Firstly, seven of the reviewed articles lacked age- and gender-matched control groups, and only one accounted for participants’ years of education. Notably, different neuropsychological assessments were used across studies, hindering result generalization. Additionally, many studies applied only one cognitive test or task to assess different memory types, necessitating the use of multiple measures for confirmation. Another limitation is the exclusive focus on memory performance in individuals with myelomeningocele-type SB; other subtypes, such as meningocele or occulta, were not included as those studies did not meet our inclusion criteria. Furthermore, most participants also had hydrocephalus and Arnold Chiari II malformation, complicating the isolation of cognitive deficits solely to SB. Although a few studies compared cognitive functioning between SB patients with and without these comorbidities, their conflicting conclusions necessitate further research. Additionally, none of the reviewed studies considered or controlled psychosocial variables, including anxious-depressive symptomatology, pain, or sleep quality. Future research should address these variables. Finally, more neuroimaging studies are required to investigate the underlying neural mechanisms supporting cognitive findings in individuals with SB.

## 5. Conclusions

The reviewed literature indicates a general consensus regarding memory impairments prevalent in SB patients. Participants in these studies commonly exhibited difficulties with short-term memory, working memory, and long-term memory (specifically, declarative memory). However, given the methodological limitations of the included studies, these findings should be interpreted cautiously.

The paucity of scientific research dedicated to the cognitive study of this clinical population significantly hinders our understanding of the relationship between memory deficits and psychological symptoms, as well as other factors such as fatigue, pain, or sleep quality, which remain unexplored in SB patients. Understanding this relationship is crucial for the effective treatment of the SB population. This knowledge gap emphasizes the necessity for future research to comprehensively examine these factors and their implications for clinical practice.

The present findings have potential implications for developing cognitive interventions aimed at mitigating or preventing memory impairments in the SB population which could ultimately enhance their functional independence and quality of life.

## Figures and Tables

**Table 1:** Search strategy according to PICOS criteria (Population; Intervention; Comparison; Outcome; Study design).

P	People with SB diagnosis. No restriction on age, cultlure.
I	Any cognitive or neuropsychological assessment on memory.
C	Any group comparison or studies using normative data for comparison.
O	Memory performance (cross-sectional studies).
S	Empirical studies (any sample size).

**Table 2:** Inclusion and exclusion criteria for the systematic review.

Inclusion Criteria	Exclusion Criteria
Reported human cases diagnosed with SB. Included participants with SB who were children, adolescents or adults. Used tests or tasks to assess memory. Used a control group for comparison. Were published in English or Spanish. Were published from 2000 until 2024.	Full-text unavailability. Book chapters, posters, or conference abstracts. Longitudinal studies. Use of self-reported measures or parent-reported measures. Single-case studies. Review articles. Lack of reported memory outcomes. Absence of a healthy control group for comparison.

**Table 3:** Search strategy.

PubMed ((spina bifida[Title/Abstract]) OR (myelomeningocele[Title/Abstract])) AND (memory[Title/Abstract])
SCOPUS TITLE-ABS (spina AND bifida OR myelomeningocele AND memory)
Web of Science ((TI=(spina bifida)) OR TI=(myelomeningocele)) AND TI=(memory) ((AB=(spina bifida)) OR AB=(myelomeningocele)) AND AB=(memory)
ProQuest title(spina bifida) OR title(myelomeningocele) AND title(memory) abstract(spina bifida) OR abstract(myelomeningocele) AND abstract(memory)

Note. TI = title; AB = abstract; ABS = abstract.

**Table 4:** Risk of bias assessment for reviewed studies.

		Methods to Control Confounding		Statistical Methods		Methods for Measuring Outcomes	
Authors, Year	Method for Selecting Sample	Sample Size	Identification of Confounders	Appropiate Analyses	Missing Data	Outcome Measures	Objective Assessment
**SB pediatric and adolescent population**							
Mammarella et al., 2003 [76]	Moderate	Moderate	Low	Low	Low	Low	Low
Burmeister et al., 2005 [63]	Low	Low	Moderate	Low	Low	Low	Low
Vachha and Adams, 2005 [75]	Moderate	Moderate	Low	Low	Low	Low	Moderate
Boyer et al., 2006 [91]	Moderate	Moderate	Low	Low	Low	Low	Low
Jansen-Osmann et al., 2008 [92]	High	High	Low	Low	Low	Low	Low
Lindquist et al., 2008 [93]	High	High	Moderate	Low	Low	Low	Low
English et al., 2010 [94]	Low	Low	Low	Low	Low	Low	Low
Hampton et al., 2013 [95]	Low	Low	Moderate	Low	Moderate	Low	Low
Raghubar et al., 2015 [60]	Low	Low	Low	Low	Low	Low	Low
Burro et al., 2018 [49]	High	High	Moderate	Low	Low	Low	Low
Attout et al., 2020 [96]	High	High	Low	Low	Low	Low	Low
Bartonek et al., 2021 [97]	Low	Low	Low	Low	Low	Low	Low
**SB adult population**							
Iddon et al., 2004 [98]	High	Moderate	Moderate	Low	Moderate	Low	Low
Dennis et al., 2007 [77]	Moderate	Moderate	Low	Low	Moderate	Low	Low
Dennis et al., 2010 [99]	Moderate	Moderate	Low	Low	Low	Low	Low
Treble-Barna et al., 2015 [86]	Low	Low	Low	Low	Low	Low	Low
Ware et al., 2017 [100]	Low	Low	Low	Low	Low	Low	Low

Note. Quality indicators were chosen based on the modified version of the Newcastle–Ottawa scale as adapted by Bawor et al. [89]. High risk of bias: High; Moderate risk of bias: Moderate; Low risk of bias: Low.

**Table 5:** Tests used to assess memory in children and adolescents with SB in the studies included in the systematic review.

Authors, Year	Test or Task Used	Subtest Applied	Type of Memory Assessed
Mammarella et al., 2003 [76]	House Visual Span	—	Visuospatial WM
	VPT	—	Visuospatial WM
	CBT	The Forward and Backward version	Visuospatial WM
Burmeister et al., 2005 [63]	WJ-R	Numbers Reversed	WM
	CVLT-C	—	Verbal memory
Vachha and Adams, 2005 [75]	Lists of 14 words with seven exemplars each of two distinct semantic categories	—	Verbal memory
Boyer et al., 2006 [91]	WISC-III	Digit Span and Arithmetic	WM
Jansen-Osmann et al., 2008 [92]	KABC	Spatial Memory	Visuospatial WM
	Virtual maze	—	Visuospatial memory
Lindquist et al., 2008 [93]	CBT	—	Visuospatial WM
	WISC	Digit Span	WM
	STORDEL	—	Verbal memory
	ROCF	—	Visuospatial memory
	SLDEL	—	Visuospatial memory
	RAVLT	—	Verbal memory
English et al., 2010 [94]	N-back test (1-back, 2-back and 3-back)	—	WM
Hampton et al., 2013 [95]	CVLT-C	—	Verbal memory
Raghubar et al., 2015 [60]	WJ-R	Numbers Reversed	WM
	WISC-IV-Integrated	Spatial Span backward	Visuospatial WM
Burro et al., 2018 [49]	WISC-IV	Digit Span and Letter-Number Sequencing	WM
Attout et al., 2020 [96]	Forward letter span	—	Short-term storage of verbal information
	Category-span task	—	Short-term storage and manipulation of verbal information
	Visuospatial span task	—	Short-term storage of visual information
Bartonek et al., 2021 [97]	CBT	—	Visuospatial WM
	WalCT	—	Topographic WM

Note. CBT = Corsi block-tapping Test; CVLT-C = California Verbal Learning Test—Children’s Version; KABC = The Kaufman-Assessment Battery for Children; RAVLT = The Rey Auditory-Verbal Learning Test; ROCF = The Complex Figure of Rey; SLDEL = The Spatial Learning Test; STORDEL = Story Recall; VPT = The Visual Patterns Test; WalCT = Walking Corsi Test; WISC = Wechsler Intelligence Scale for Children; WISC-III = Wechsler Intelligence Scale for Children—Third Edition; WISC-IV = The Wechsler Intelligence Scale for Children—Fourth edition; WISC-IV-Integrated = The Wechsler Intelligence Scale for Children—Fourth Edition Integrated; WM = working memory; WJ-R = The Woodcock Johnson Test of Cognitive Abilities-Revised.

**Table 6:** Tests used to assess memory in adults with SB in the studies included in the systematic review.

Authors, Year	Test or Task Used	Subtest Applied	Type of Memory Assessed
Iddon et al., 2004 [98]	CANTAB	-	Visual and spatial memory, spatial memory span and spatial WM
	HVLT	-	Verbal memory (immediate recall, learning, recognition and delayed recall)
Dennis et al., 2007 [77]	RBMT-E	Belongings, Appointments, Messages and Immediate and Delayed Story	Prospective memory and verbal episodic memory (recall)
	The MicroCog^TM^ computerized test	Numbers forward, Numbers reversed, Tic Tac, Story (immediate) 1 and 2, Story (delayed) 1 and 2, Wordlist 1, Wordlist 2, Address and Timers	WM (maintenance and manipulation), immediate and delayed verbal episodic memory (recognition), semantic memory and non-declarative memory
	TEA	Lottery and Elevator Counting with Reversal	WM
Dennis et al., 2010 [99]	CAMPTROMPT	-	Prospective memory
Treble-Barna et al., 2015 [86]	RBMT-E	Belongings, Appointments, Messages and Immediate and Delayed Story	Prospective memory and verbal episodic memory (recall)
Ware et al., 2017 [100]	WMST and WMMT	-	Visual WM

Note. CANTAB = The Cambridge Neuropsychological Test Automated Battery; CAMPTROMPT = The Cambridge Prospective Memory Test; HVLT = The Hopkins Verbal Learning Test; RBMT-E = Rivermead Behavioral Memory Test—Extended Version; TEA = The Test of Everyday Attention; WM = working memory; WMMT = Working Memory Manipulation Task; WMST = Working Memory Span Task.

**Table 7:** Studies conducted on pediatric and adolescent population with SB that assessed memory.

Authors, Year	Sample (*N*)	Demographic Variables		Results
		Age (M/SD) (min.–max.)	Sex (n/%)	
Mammarella et al., 2003 [76]	*N* = 40 **Clinical group** (SB): *N* = 20 **Control group** (TD): *N* = 20	**SB group:** 10.5 (-) **TD group:** 10.5 (-) (8–13 years)	**SB group:** F = 11 (55%) M = 9 (45%) **TD group:** F = 10 (50%) M = 10 (50%)	Statistically significant differences were observed in the performance of the House Visual Span task (*p* < 0.01) between the clinical SB group (*M* = 27.30; *SD* = 2.79) and the control group (*M* = 29.85; *SD* = 1.98). However, no differences were observed in the rest of the visuospatial WM tests and tasks used (*p* > 0.05).
Burmeister et al., 2005 [63]	*N* = 205 **Clinical group** (SB): *N* = 164 Non-ADHD: *N* = 112 ADHD-I: *N* = 38 ADHD-C: *N* = 14 **Control group** (TD): *N* = 41	(Age by months) **SB group:** Non-ADHD: 129.7 (30.8) ADHD-I: 142.0 (27.0) ADHD-C: 119.0 (22.0) **TD group:** 139.2 (33.8) (7–16 years)	**SB group:** Non-ADHD: F = 60 (54%) M = 52 (46%) ADHD-I: F = 19 (50%) M = 19 (50%) ADHD-C: F = 5 (36%) M = 9 (64%) **TD group:** F = 22 (54%) M = 19 (46%)	No differences were observed in the performance of the different cognitive tasks in children with SB when divided into groups based on the ADHD classification. The performance on the “Numbers Reversed” subtest, that assessed WM, was significantly poorer in children with SB compared to the TD participants (*p* < 0.05). The performance on the California Verbal Learning Test—Children’s Version, that assessed verbal memory, was significantly poorer in children with SB compared to the TD participants (*p* < 0.0001)
Vachha and Adams, 2005 [75]	*N* = 52 **Clinical group** (SB): *N* = 26 **Control group** (TD): *N* = 26	**SB group:** 12.3 (2.7) **TD group:** 11.2 (2.6) (7–16 years)	-	The mean memory span was significantly lower across the three trials in participants with SB compared to the TD participants (*p* < 0.001). In this way, children with SB remembered fewer words than their healthy peers.
Boyer et al., 2006 [91]	*N =* 58 **Clinical group** (SB): *N* = 31 **Control group** (TD): *N* = 27	**SB group:** 10.68 (2.23) **TD group:** 12.26 (2.09) (8–15 years)	**SB group:** F = 9 (29%) M = 22 (71%) **TD group:** F = 12 (44%) M = 15 (56%)	Significant differences were found in the score obtained by the SB group compared to the TD group in one of the WM tasks *(*“Arithmetic”) (*p* < 0.001) but not for the other one (“Digit Span”) (*M* = 9.19; *SD* = 2.63 in the SB group and *M* = 10.07; *SD* = 2.35 in the control group).
Jansen-Osmann et al., 2008 [92]	*N* = 40 **Clinical group** (SB): *N* = 20 **Control group** (TD): *N* = 20	**SB group:** 11.4 (1.7) **TD group:** 11.8 (1.8) (8–14 years)	**SB group:** F = 13 (65%) M = 7 (35%) **TD group:** F = 13 (65%) M = 7 (35%)	The SB clinical group (*M* = 14.40; *SE* = 3.50) performed worse on the virtual visuospatial WM task compared to their healthy peers (*M* = 16.90; *SE* = 2.49) (*p* < 0.05). A significant correlation was found between the age of walking and the score obtained in the visuospatial WM task, measured by number of points (*p* < 0.01) and number of learning trials during the performance on the maze of the task (*p* < 0.01). In this way, children in the clinical SB group that learned to walk later in life obtained fewer points on the visuospatial WM test, needing more trials to figure out the correct path in the virtual maze.
Lindquist et al., 2008 [93]	*N* = 72 **Clinical group 1** (SB): *N* = 16 **Clinical group 2** (H): *N* = 20 **Control group** (TD): *N* = 36	**SB group:** 11 years and 7 months (-) **H group:** 11 years and 7 months (-) **TD group:** 11 years and 7 months (-) (8–13 years)	**SB group:** - **H group:** - **TD group:** F = 13 (36.1%) M = 23 (63.9%)	Participants with SB obtained significantly lower results on the following tasks measuring short-term memory compared to the healthy participants: Story Recall and The Complex Figure of Rey, (1.5 SD under test norm on both tests). Similar results were found, showing a worse performance in the SB group compared to their healthy peers in the Story Recall and Rey Auditory Verbal Learning tests (1.5 SD under test norm on both tests). The SB clinical group’s performance was significantly inferior compared to their healthy peers on the two visuospatial memory tasks (The Complex Figure of Rey and *The Spatial Learning Test*) (*p* < 0.01). However, there were no significant differences in the performance of the “Corsi block test” and “Digit Span” subtest. The SB and H groups did not differ in their performance on the different cognitive tasks.
English et al., 2010 [94]	*N* = 118 **Clinical group** (SB): *N* = 79 **Control group** (TD): *N* = 39	**SB group:** 12.5 (2.7) **TD group:** 12.3 (2.8) (8–19 years)	**SB group:** F = 38 (48.10%) M = 41 (51.90%) **TD group:** F = 21 (53.85%) M = 18 (46.15%)	A lower WM score was observed in the SB clinical group (*M* = 27.6; *SD* = 11.9) compared to the TD control group (*M* = 37.8; *SD* = 9.9) (*p* < 0.001). Thus, the SB group showed less developed WM abilities compared to the TD group.
Hampton et al., 2013 [95]	*N =* 180 **Clinical group 1** (SB): *N* = 151 **Clinical group 2** (AS): *N* = 29 **Control group** (TD): *N* = 60	**SB group:** 11.37 (2.80) **AS group:** 12.52 (3.30) **TD group:** 12.08 (2.84) (7–18 years)	**SB group:** F = 66 (44%) M = 85 (56%) **AS group:** F = 13 (45%) M = 16 (55%) **TD group:** F = 31 (52%) M = 29 (48%)	The SB clinical group had the lowest average score on verbal memory compared to the clinical AS group and to the TD group. The clinical SB group and the clinical AS group differed significantly in performance in the verbal memory domain (*p* < 0.05)
Raghubar et al., 2015 [60]	*N* = 94 **Clinical group** (SB): *N* = 44 **Control group** (TD): *N* = 50	**SB group:** 9.91 (-) **TD group:** 9.81 (not specified)	**SB group:** F = - (63%) M = - (37%) **TD group:** F = - (43%) M = - (57%)	A poorer performance on measures of verbal (*p* = 0.01) and visuospatial WM (*p* = 0.001) was observed in the SB clinical group when compared to the TD group.
Burro et al., 2018 [49]	*N* = 26 **Clinical group** (SB): *N* = 13 **Control group** (TD): *N* = 13	**SB group:** 12.85 (2.91) **TD group:** 12.85 (2.91) (7.6–16.0 years)	**SB group:** F = 5 (38.5%) M = 8 (61.5%) **TD group:** F = 5 (38.5%) M = 8 (61.5%)	The general performance in the WM Index was lower in the SB clinical group than in the TD group. A significantly lower performance was observed in the SB group compared to the TD group on the “Letter-Number Sequencing” subtest (*p* < 0.001). However, no statistically significant differences were found between the SB and the TD groups on the “Digit Span” subtest (*M* = 7.92; *SD* = 3.33 and *M* = 10.54; *SD* = 3.78, respectively).
Attout et al., 2020 [96]	*N =* 46 **Clinical group** (SB): *N* = 23 **Control group** (TD): *N* = 23	(Age by months) **SB group:** 145.26 (28.46) **TD group:** 145.65 (28.03) (7–16 years)	**SB group:** F = 10 (43.48%) M = 13 (56.52%) **TD group:** F = 12 (52.17%) M = 11 (47.82%)	Lower capacities in verbal (*p* < 0.001) and visuospatial WM (*p* < 0.001) were found in the SB group compared to the TD group. However, no differences were found between the SB and the TD groups in the performance on the Forward Letter Span Task (*p* = 0.14).
Bartonek et al., 2021 [97]	*N* = 161 **Clinical group 1** (SB): *N* = 41 **Clinical group 2** (AMC): *N* = 10 **Control group** (TD): *N* = 120	**SB group:** 11.9 (3.2) **AMC group:** 10.6 (3.1) **TD group:** 9.9 (3.1) -	**SB and AMC group:** F= 19 (-) M = 22 (-) **TD group:** F = 63 (52.5%) M = 57 (47.5%)	The score obtained in WalCT that measured topographic WM was lower in the SB group compared to the control group. No differences were found between the scores obtained in the SB group and the AMC and TD groups on the CBT visuospatial memory test. However, a significant difference was found between the non-ambulation group (which was conformed by only children with SB that had to use a wheelchair for all their transfers) and the TD group on the CBT span (*p* = 0.004). In this way, non-ambulant SB children obtained the lowest scores on visuospatial WM, showing a poorer visuospatial function than those SB children who could walk independently.

Note. ADHD-I *=* attention deficit/hyperactivity disorder inattentive type; ADHD-I *=* attention deficit/hyperactivity disorder combined type; AMC *=* arthrogryposis multiplex congenita; AS = aqueductal stenosis; CBT *=* Corsi block-tapping test; F = female; M = male or mean; Non-ADHD *=* no diagnosis of ADHD; *p* = *p*-value; *SB* = spina bifida; SD = standard deviation; SE *=* standard error; TD = typically developed; WalCT = walking Corsi test; WM = working memory.

**Table 8:** Studies conducted on adult population with SB that assessed memory.

Authors, Year	Sample (*N*)	Demographic Variables		Results
		Age (M/SD) (min.–max.)	Sex (n/%)	
Iddon et al., 2004 [98]	*N* = not specified. Ranged from 72 to 159 in memory measures. **Clinical group 1** (SB with concomitant hydrocephalus) **Clinical group 2** (H) **Clinical group 3** (SB alone without concomitant hydrocephalus) **Control group** (TD)	-	-	Statistically significant differences were observed between the clinical groups and the control group (or normative data) on every score from the CANTAB and the Hopkins Verbal Learning Test. SB participants with a hydrocephalus diagnosis obtained lower scores in all the memory tasks compared to participants with SB alone (without a hydrocephalus diagnosis) and the TD group. The majority of the SB group’s results were average or above average. These results suggest that individuals with SB without hydrocephalus do not show the same memory impairments as those with SB and hydrocephalus.
Dennis et al., 2007 [77]	*N* = 58 **Clinical group** (SB): *N* = 29 0–5 shunt revisions: *N* = 17 >5 revisions: *N* = 12 **Control group** (TD): *N* = 29	**SB group:** 26.60 (4.82) 0–5 shunt revisions: 27.53 (4.83) >5 revisions: 25.28 (4.69) **TD group:** 26.88 (5.86) (18.21–36.50 years)	**SB group:** F = 13 (44.83%) M = 16 (55.17%) **TD group:** F = 18 (62.07%) M = 11 (37.93%)	SB group performance differed significantly from that of the TD group on prospective memory tasks (*p* < 0.05). Within the SB group, patients with more than five shunt revisions showed poorer performance than healthy controls (*p* < 0.02). Verbal episodic memory tasks included recall and recognition (both immediate and delayed). In recognition tasks, the SB group performed similarly to normative data on immediate recall, but scored lower than the population mean on delayed tasks (*p* < 0.03), regardless of shunt revisions. In recall tasks, the SB group differed from the TD group on both immediate (*p* < 0.05) and delayed recall (*p* < 0.01). However, only those with more than five shunt revisions scored significantly lower than the TD group on the immediate recall task (*p* < 0.02). The SB group performed significantly worse than the population mean on WM tasks, regardless of shunt revisions. This included a maintenance task (*p* < 0.01) and a maintenance/manipulation task (*p* < 0.03). However, on another maintenance task, only those with fewer shunt revisions scored lower than normative means on another maintenance task. No significant differences were found on inhibition and set-shifting tasks. Participants with SB and fewer than six revisions scored higher than population means on the non-declarative memory task (*p* < 0.03). The SB group showed no significant difference from normative data on the semantic memory task (*p* > 0.05).
Dennis et al., 2010 [99]	*N* = 49 **Clinical group** (SB): *N* = 32 **Control group** (TD): *N* = 17	**SB group:** 34.48 (10.34) **TD group:** 30.29 (14.94) (18.33–62.67 years)	**SB group:** F = 18 (56.25%) M = 14 (43.75%) **TD group:** F = 12 (70.59%) M = 5 (29.41%)	Differences were observed on the performance on the prospective memory test between the SB clinical group and the control group in the total score (*p* < 0.0001), time-based subscore (*p* < 0.0001) and event-based subscore (*p* < 0.001). Twenty-five percent of the participants in the SB group showed an impaired prospective memory. The older adults in the SB group had a threefold higher rate of poor prospective memory compared to the younger adults in the SB group (37.50% and 12.50%, respectively) (*p* < 0.05).
Treble-Barna et al., 2015 [86]	*N* = 138 **Clinical group** (SB): *N* = 97 **Control group** (TD): *N* = 41	**SB group:** 29.14 (9.7) **TD group:** 30.45 (11.9) (18–62 years)	**SB group:** F = 52 (54%) M = 45 (46%) **TD group:** F = 29 (71%) M = 12 (29%)	Prospective memory performance was significantly lower in participants with SB (*M* = 18.57; *SE* = 0.36) compared to the TD participants (*M* = 21.92; *SE* = 0.55). Verbal episodic memory performance was significantly lower in participants with SB (*M* = 9.41; *SE* = 0.62) compared to the TD participants (*M* = 15.50; *SE* = 0.95).
Ware et al., 2017 [100]	*N =* 120 **Clinical group** (SB): *N* = 68 **Control group** (TD): *N* = 52	**SB group:** 28.61 (9.71) **TD group:** 31.81 (10.48) (18–56 years)	**SB group:** F = 33 (49%) M = 35 (51%) **TD group:** F = 38 (73%) M = 14 (27%)	Regarding the WMST, the performance accuracy was significantly lower in the SB group compared to the TD group (*p* < 0.001). Older and younger adults from the SB group presented a similar performance accuracy (*p* = 0.301). In contrast, older adults in the TD group had a significantly poorer performance on accuracy (*p* < 0.05). These findings elucidate a general impaired profile that seem to be maintained across aging in adults with SB. Reaction time was similar for both SBM and TD groups (*p* = 0.740). Regarding the WMMT, the performance accuracy was significantly lower in the SB group compared to the TD group (*p* = 0.002). Reaction time was similar for both SB and TD groups (*p* > 0.05).

Note. CANTAB = The Cambridge Neuropsychological Test Automated Battery; H = isolated hydrocephalus; F = female; M = male or mean; *p* = *p*-value; SB = spina bifida; SD = standard deviation; SE = standard error; TD = typically developed; WM = working memory; WMMT = Working Memory Manipulation Task; WMST = Working Memory Span Task.
